# Greater Social Engagement and Greater Gray Matter Microstructural Integrity of Aging Adults

**DOI:** 10.1093/geroni/igaa057.2879

**Published:** 2020-12-16

**Authors:** Cynthia Felix, Caterina Rosano, Xiaonan Zhu, Jason Flatt, Andrea Rosso

**Affiliations:** 1 University of Pittsburgh, Pittsburgh, Pennsylvania, United States; 2 University of Nevada, Las Vegas, Las Vegas, Nevada, United States

## Abstract

Social engagement reflects habitual social roles in aging adults and may protect against dementia. Cross-sectional associations of social engagement (SE) index with gray matter (GM) microstructure was studied in regions of interest relevant to social cognition among community-dwelling older adults [n=293, mean age: 82.8 years (SD: 2.8), 43% males] using linear regression models. Greater SE was significantly related to lower mean diffusivity (MD) (greater GM microstructural integrity) [shown as standardized estimate (p-value)] in: left middle frontal gyrus-orbital part: -0.168 (0.005), left caudate nucleus: -0.141 (0.02), left temporal pole-middle temporal gyrus: -0.136 (0.03), right middle frontal gyrus: -0.160 (0.006), right superior frontal gyrus-orbital part: -0.187 (0.002), right middle frontal gyrus, orbital part: -0.124 (0.04), adjusted for demographic attributes. Associations were robust to adjustment for hearing or ADL difficulty. Findings were generally stronger in females than in males. Social engagement may prevent GM integrity loss and build brain reserve in dementia-related regions. Part of a symposium sponsored by Brain Interest Group.

